# Draft Genome Sequence of Pseudomonas protegens Strain MWU12-2233, Isolated from Wild Cranberry Fruit in Provincetown, Massachusetts

**DOI:** 10.1128/mra.00546-22

**Published:** 2022-07-18

**Authors:** Michelle Wood, Scott D. Soby

**Affiliations:** a Biomedical Sciences, College of Graduate Studies, Midwestern University, Glendale, Arizona, USA; b College of Veterinary Medicine, Midwestern University, Glendale, Arizona, USA; University of Arizona

## Abstract

Pseudomonas protegens strain MWU12-2233 was isolated from wild cranberry fruit surfaces in Provincetown, MA. The genome contains putative hydrogen cyanide synthase and type VI secretion systems which can act symbiotically on plant health by suppressing competitors, indicating a role in indigenous microfloral disease and insect pest suppression.

## ANNOUNCEMENT

Protected wetlands represent an understudied ecosystem that harbors diverse communities of phyllosphere bacteria, including those associated with wild cranberry flowers and fruits (1, 2). Some of these bacteria are pseudomonads that produce secondary metabolites with biological activity against fungi and insects (3), including the plant-growth-promoting and insectivorous bacterium Pseudomonas protegens (4–7), potentially making them a component of the indigenous disease-suppressive microflora. P. protegens MWU12-2233 was isolated in July 2012 from cranberry fruit as part of a culture-dependent survey of bacteria from wild cranberry bogs in the Cape Cod National Seashore, Provincetown, MA (42.070624 N, 70.210548 W). Plant phenology at the time of sampling was late flowering to early fruit set. Cranberry fruits were vortexed in sterile water, and the water was plated on King’s medium B (KMB) agar containing 50 μg · mL^−1^ each of ampicillin and cycloheximide. Colonies were picked for isolation onto fresh KMB if they fluoresced under long-wave UV light, single-colony purified 3 times, and stored at −80°C in 34% glycerol. MWU12-2233 was placed initially in the genus Pseudomonas by a 16S rRNA gene sequence amplified with 27F and 1525R primers, using BLAST (8) within the NCBI nucleotide database. Genomic DNA was isolated with a DNeasy blood and tissue kit (Qiagen) from overnight KMB broth cultures, and libraries were generated with the Kapa Biosystem Hyperplus library preparation kit (KK8514). DNA was enzymatically fragmented to approximately 500 bp, end repaired, and A-tailed as described in the Kapa protocol. Illumina-compatible adapters with unique indexes (Integrated DNA Technologies; 00989130v2) were ligated individually to each sample, followed by cleaning with Kapa pure beads (Kapa Biosciences; KK8002), and amplified with a HiFi enzyme (KK2502). Fragment size was determined on an Agilent Tapestation system and quantified using quantitative PCR (qPCR) (Kapa library quantification kit, KK4835) on a ThermoFisher Quantstudio 5 instrument. The library was multiplex pooled for sequencing on an Illumina MiSeq platform in a 2 × 250 bp flow cell. Raw reads were assembled and quality controlled in the PATRIC (http://patricbrc.org) Comprehensive Genome Analysis pipeline v3.6.12 using Unicycler v0.4.8 and two rounds of polishing with Pilon v1.23 using default settings except for the automated trimming function, which was set to “true” (9–11). The pipeline includes quality control with Trim Galore v0.4.0 (https://www.bioinformatics.babraham.ac.uk/projects/trim_galore/) (12). P. protegens MWU12-2233 had a genome size of 6,586,881 bp assembled into 23 contigs, from 1,228,931 reads, and a total read length of 581,147,449 bp. The G+C content was 63.4% and *N*_50_ value was 806,444 bp with 88× coverage. The isolate was identified as P. protegens by Genome BLAST distance phylogeny approach (GBDP) using the type strain genome server (TYGS) online tool (https://tygs.dsmz.de/) (13). MWU12-2233 contains HCN synthase genes, as do other isolates of P. protegens (14, 15), as well as a type VI secretion system (16), of which both may be part of a suite of functions that makes MWU12-2233 a disease-suppressive component of the cranberry phytobiome.

### Data availability.

This whole-genome shotgun project has been deposited at DDBJ/EMBL/GenBank under the accession number JALHAR000000000 for P. protegens MWU12-2233. The version described in this paper is the first version, JALHAR000000000.1, BioProject PRJNA691338, and BioSample SAMN26894067. The Sequence Read Archive accession number is SRR18508440. RASTtk annotations are available under open license at Zenodo (https://zenodo.org/record/6392145#.YlRrdsjMK3A).
